# Improved Visible-Light Photocatalytic H_2_ Evolution of G-C_3_N_4_ Nanosheets by Constructing Heterojunctions with Nano-Sized Poly(3-Thiophenecarboxylic Acid) and Coordinating Fe(III)

**DOI:** 10.3390/nano13081338

**Published:** 2023-04-12

**Authors:** Yong Li, Bingmiao Zhang, Xulong Pang, Zhijun Li, Yi Zhang, Ming Hao, Yan Zhu, Chuanli Qin, Liqiang Jing

**Affiliations:** 1 Key Laboratory of Functional Inorganic Material Chemistry (Ministry of Education), School of Chemistry and Materials Science, Heilongjiang University, Harbin 150080, China; 2 Engineering Research Center for Hemp and Product in Cold Region of Ministry of Education, Qiqihar University, Qiqihar 161006, China

**Keywords:** graphitic carbon nitride, poly(3-thiophenecarboxylic acid) modification, coordinated Fe(III), charge transfer and separation, photocatalytic hydrogen evolution

## Abstract

It is highly desirable to enhance the photogenerated charge separation of g-C_3_N_4_ by constructing efficient heterojunctions, especially with an additional organic constitution for solar–hydrogen conversion. Herein, g-C_3_N_4_ nanosheets have been modified controllably with nano-sized poly(3-thiophenecarboxylic acid) (PTA) through in situ photopolymerization and then coordinated with Fe(III) via the -COOH groups of modified PTA, forming an interface of tightly contacted nanoheterojunctions between the Fe(III)-coordinated PTA and g-C_3_N_4_. The resulting ratio-optimized nanoheterojunction displays a ~4.6-fold enhancement of the visible-light photocatalytic H_2_ evolution activity compared to bare g-C_3_N_4_. Based on the surface photovoltage spectra, measurements of the amount of •OH produced, photoluminescence (PL) spectra, photoelectrochemical curves, and single-wavelength photocurrent action spectra, it was confirmed that the improved photoactivity of g-C_3_N_4_ is attributed to the significantly promoted charge separation by the transfer of high-energy electrons from the lowest unoccupied molecular orbital (LUMO) of g-C_3_N_4_ to the modified PTA via the formed tight interface, dependent on the hydrogen bond interaction between the -COOH of PTA and the -NH_2_ of g-C_3_N_4_, and the continuous transfer to the coordinated Fe(III) with -OH favorable for connection with Pt as the cocatalyst. This study demonstrates a feasible strategy for solar-light-driven energy production over the large family of g-C_3_N_4_ heterojunction photocatalysts with exceptional visible-light activities.

## 1. Introduction

Hydrogen is a potential energy carrier that is high energy density and carbon-free, making it a splendid candidate for energy storage and conversion. Photocatalytic water splitting is one of the simplest pathways to mimic photosynthesis for the conversion of solar energy into storable, clean and renewable H_2_ fuels, which can well relieve the pressure caused by environmental and energy problems [1,2]. It is well-known that in the clean and environmentally friendly photocatalytic process, a semiconductor photocatalyst is excited by light to generate electrons and holes so as to induce reduction and oxidation reactions. Thus, the semiconductor photocatalyst is the key factor affecting the photocatalytic reaction [3]. Since visible light comprises large proportion (approximately 44%) of the sunlight that reaches the Earth’s surface, from the perspective of practical applications, it is of great significance to develop photocatalysts that can respond in the wide visible range. For this reason researchers have conducted significant research and exploration in the field of photocatalysts with a visible-light response [4,5,6].

Compared with inorganic semiconductors, organic polymer semiconductors have attracted an increasing amount of attention from researchers as promising photocatalysts due to their inherent advantages, such as their easily tunable band gap structure, metal-free nature, nontoxicity and Earth-abundant elemental compositions. Among various polymer photocatalysts investigated in the past decade, graphitic carbon nitride (g-C_3_N_4_) has become a research hotspot in the field of photocatalysis as a non-toxic, metal-free photocatalyst due to its high thermal stability, facile synthesis process and low-cost materials [7]. G-C_3_N_4_ can respond to visible light, and its photogenerated electrons possess enough energy to induce the water reduction reaction for H_2_ evolution thermodynamically due to its appropriate band gap (2.7 eV) and lowest unoccupied molecular orbital (LUMO) energy level (about −3 eV vs. vacuum level) [8]. Therefore, g-C_3_N_4_ is widely used in photocatalytic H_2_ evolution [3,4], CO_2_ reduction [9,10] and so on. However, it has been found that g-C_3_N_4_ suffers from fast photogenerated charge recombination and a narrow visible-light response range, resulting in its relatively low photocatalytic H_2_ evolution activities. Researchers tried to modify g-C_3_N_4_ by integrating it with other photocatalytic materials, such as metal oxides [11,12], 2D materials [13,14,15,16] and carbon nanostructures [17,18]. Among the various modification strategies investigated, constructing heterojunctions has proven to be one of the most promising ways to enhance photogenerated charge separation and thus improve photocatalytic activities. Based on the above-mentioned advantages of polymer semiconductors, constructing g-C_3_N_4_ heterojunctions with another polymer semiconductor is regarded as an effective strategy [19,20,21,22]. Bai et al. reported poly(3-hexylthiophene)/g-C_3_N_4_ (P_3_HT-g-C_3_N_4_) p-n heterojunctions achieved using a ball milling method, and the photocatalytic activity of the P_3_HT-g-C_3_N_4_ photocatalysts for the degradation of MB was two times higher than that of pure g-C_3_N_4_ [23]. Miao et al. constructed perylenetetracarboxylic diimide/g-C_3_N_4_ (PDI/g-C_3_N_4_) 1D/2D heterojunctions in which the Z-scheme pathway of the carriers led to the spatial separation of redox reaction sites and a 2.03-fold enhancement of the photocatalytic H_2_ evolution activity compared to g-C_3_N_4_ [24]. However, the interface contact was not considered in these works. It is well-known that the electronic transfer mechanism of polymer semiconductors is slightly different from that of inorganic semiconductors. Additionally, the dielectric constant of polymer semiconductors is low, so their photogenerated charge binding energy is large, resulting in unfavorable spontaneous dissociation [25]. It is worth mentioning that weak interface contacts between g-C_3_N_4_ and another polymer semiconductor could result in unsatisfactory photocatalytic performances of polymer semiconductor/g-C_3_N_4_ heterojunctions [8,23,24,26]. Therefore, tight interface contact in polymer/g-C_3_N_4_ heterojunctions is the key to achieving excellent photocatalytic performance. Since some amino groups (-NH_2_) exist on g-C_3_N_4_, synthesized by the thermal condensation of monomers (such as urea, melamine and so on), it is practical to introduce another polymer semiconductor with abundant carboxy groups (-COOH) to effectively connect to the g-C_3_N_4_ via hydrogen bonding [27,28]. Therefore, it is particularly critical to select a suitable polymer semiconductor with abundant -COOH to fabricate g-C_3_N_4_ heterojunctions with a tight interface contact via hydrogen bonding interaction to promote photogenerated charge separation and thus enhance the visible-light catalytic H_2_ evolution activities.

As a polythiophene derivative, poly(3-thiophenecarboxylic acid), with abundant hydrophilic -COOH on the main chain, has high electrical conductivity and good environmental stability and is cost-effective. Additionally, PTA is a promising polymer photocatalyst for H_2_ evolution with a wide visible-light response because PTA has a narrow energy gap (1.9–2.1 eV) and an appropriate LUMO energy level (about −3.2 eV vs. vacuum level) [29], meeting the thermodynamic standard of reducing water to produce H_2_. More importantly, PTA makes it possible to fabricate the tightly contacted heterojunctions with g-C_3_N_4_ through the hydrogen bond interaction between the -COOH of PTA and the -NH_2_ of g-C_3_N_4_, which is favorable for promoting the photogenerated charge transfer and separation. In addition, PTA displays excellent water dispersibility since it has a hydrophilic -COOH in each of the repeating units, increasing the opportunity for reaction collisions with H_2_O molecules and thus enhancing the photocatalytic H_2_O reduction performance for H_2_ evolution [30,31]. Inspired by these factors, it is highly anticipated that PTA will be introduced to construct tightly contacted heterojunctions with g-C_3_N_4_ for promoting the photogenerated charge transfer and separation, extending the visible-light response, improving the water dispersibility and thus enhancing the visible-light photocatalytic H_2_ evolution activities.

For photocatalysts, the coordinated transition metal ions play an important role in their photogenerated charge transfer and separation. Sardar et al. revealed an efficient photoinduced electron-transfer processes in Fe(III)-hematoporphyrin-TiO_2_ nanohybrids from excited hematoporphyrin to Fe(III), which was responsible for the improved photocatalytic performance [32]. Zhang et al. proved that the photogenerated electrons of g-C_3_N_4_ transferred to tetra(4-carboxyphenyl)porphyrin iron(III) chloride (FeTCPP), leading to the formation of Fe^II^TCPP, [Fe^II^TCPP…CO_2_], [Fe^I^TCPP…CO_2_]^−^ and [Fe^0^TCPP…CO_2_]^2−^ intermediates in g-C_3_N_4_/FeTCPP heterogeneous catalysts upon irradiation, thus the improved photocatalytic activity for CO_2_ reduction [28]. As one of the interesting coordinating moieties, -COOH is widely employed for the construction of transition metal carboxylate clusters by coordinating with transition metal ions, using either one or both oxygen atoms [33,34]. Based on the above considerations, it is highly feasible to construct Fe(III)-coordinated PTA/g-C_3_N_4_ heterojunctions by coordinating Fe(III) with the -COOH of PTA. This could improve the photogenerated charge transfer and separation and thus the visible-light photocatalytic activities. As far as we know, Fe(III)-coordinated PTA/g-C_3_N_4_ heterojunctions for visible-light photocatalytic H_2_ evolution have not been reported.

In this work, PTA/g-C_3_N_4_ nanoheterojunctions with tight interface contact were first successfully synthesized using the in situ photopolymerization method. Fe(III)-coordinated PTA/g-C_3_N_4_ nanoheterojunctions were then constructed using the simple impregnation method with an Fe(NO_3_)_3_ solution. It was proven that the tight interface contact, dependent on the hydrogen bond interaction between the -COOH of PTA and the -NH_2_ of g-C_3_N_4_, is responsible for the promoted photogenerated charge separation and thus the enhanced visible-light catalytic H_2_ evolution activities and the visible photosensitization of the PTA. Interestingly, by coordinating with the -COOH of PTA, the introduced Fe(III) ions further improve the photogenerated charge separation and visible-light catalytic H_2_ evolution activities. By rational optimization, the resulting 0.5Fe-2PTA/g-C_3_N_4_ shows H_2_ evolution activity of up to 687.3 µmol h^−1^ g^−1^ under irradiation with visible light, which is approximately a ~4.6-fold enhancement of g-C_3_N_4_. Moreover, single-wavelength photocurrent action spectra illustrate that the promoted charge separation is mainly attributed to the high-level electron transfer from the LUMO of g-C_3_N_4_ to the LUMO of PTA. This work offers a practicable route to design and fabricate visible-light-driven g-C_3_N_4_ heterojunction photocatalysts for solar–hydrogen conversion.

## 2. Materials and Methods

### 2.1. Synthesis of G-C_3_N_4_ Nanosheets

G-C_3_N_4_ nanosheets were synthesized according to our previous report [35]. Typically, a certain amount of urea was placed into an alumina combustion boat with a cover, and was then heated to 550 °C for 3 h at a rate of 0.5 °C min^−1^ in a muffle furnace in an air atmosphere. After being cooled to room temperature naturally, the light-yellow product was collected and ground into powder.

### 2.2. Synthesis of PTA/g-C_3_N_4_ Nanoheterojunctions

PTA/g-C_3_N_4_ nanoheterojunctions were synthesized by the in situ photopolymerization method, according to the literature [36,37]. A certain amount of g-C_3_N_4_ and 3-thiophenecarboxylic acid (TA) was dispersed in 150 mL deionized water, stirred for 1 h and then treated by ultrasonication for 30 min. The potassium dichromate (K_2_Cr_2_O_7_), with a 1:8 molar ratio of TA to K_2_Cr_2_O_7_, was then added into the above suspension with stirring for another 1 h. The mixture was irradiated under visible light (*λ* > 420 nm) for 48 h in a cooled water bath. The PTA/g-C_3_N_4_ nanoheterojunctions, obtained by filtering, were washed with 1 M HCl and deionized water three times, dried overnight at 60 °C in a vacuum and ground into powder for further use. The obtained nanoheterojunctions were denoted as xPTA/g-C_3_N_4_, where x represents the mass percent of PTA in the PTA/g-C_3_N_4_ nanoheterojunctions (x = 1, 2, 3 and 5 wt%). For comparison, the PTA was prepared by the photopolymerization without g-C_3_N_4_ under the same other conditions.

### 2.3. Synthesis of Fe(III)-Coordinated PTA/g-C_3_N_4_ Nanoheterojunctions

Fe(III)-coordinated PTA/g-C_3_N_4_ nanoheterojunctions were fabricated using a simple impregnation method. In a typical procedure, a certain amount of 2PTA/g-C_3_N_4_ was dispersed in 100 mL aqueous solution with a certain amount of Fe(NO_3_)_3_·9H_2_O as the iron source, stirred for 48 h, filtered and then dried at 60 °C overnight in a vacuum. The samples were ground into powder and denoted as yFe-2PTA/g-C_3_N_4_ for further use, where y represents the mass percent of the Fe(III) in the yFe-2PTA/g-C_3_N_4_ nanoheterojunctions (y = 0.1, 0.3, 0.5, 0.8 and 1.0 wt%). For comparison, 0.5Fe-g-C_3_N_4_ and 0.5Fe-PTA with 0.5 wt% Fe(III) in the samples were prepared through the same impregnation method without PTA or g-C_3_N_4_ under the same other conditions, respectively.

For comparison, Fe(III)-polythiophene/g-C_3_N_4_ heterojunctions were fabricated in which there was no -COOH on the main chain of polythiophene (PTh), so there was no hydrogen bond or coordination bond interaction between the PTh and g-C_3_N_4_ or Fe(III). Firstly, 2PTh/g-C_3_N_4_ was synthesized through the in situ chemical oxidation method. Typically, 980 mg g-C_3_N_4_ was dispersed in 150 mL acetonitrile solution, stirred for 1 h and then treated by ultrasonication for 30 min. A total of 19.1 μL thiophene was added into the above suspension with stirring for another 1 h, and then 5 mL acetonitrile solution containing 77.1 mg ferric chloride was added into the suspension dropwise and stirred in an ice bath for 3 h. The 2PTh/g-C_3_N_4_, obtained by filtering, was washed with anhydrous methanol and deionized water three times and then dried overnight at 60 °C in a vacuum. Secondly, 0.5Fe-2PTh/g-C_3_N_4_ was prepared using the same impregnation method. In detail, 300 mg 2PTh/g-C_3_N_4_ was dispersed in 100 mL aqueous solution containing 10.8 mg Fe(NO_3_)_3_·9H_2_O, stirred for 48 h, filtered and then dried overnight at 60 °C in a vacuum. The obtained sample was ground into powder and denoted as 0.5Fe-2PTh/g-C_3_N_4_.

### 2.4. Evaluation of Photocatalytic Activities for H_2_ Evolution

The H_2_ evolution measurements were carried out in a 250 mL quartz cell with a 300 W Xe lamp with a cut-off filter (*λ* > 420 nm). Typically, 100 mg sample was added into the reactor, in addition to 100 mL of 10 vol% triethanolamine (TEOA) aqueous solution, which acted as the cavitation scavenger. Before irradiation, the system was vacuumed to remove dissolved air. Then, 1 wt% Pt was loaded onto the surface of the photocatalyst as a cocatalyst by the in situ photodeposition of H_2_PtCl_6_·6H_2_O for 2 h. The sample was then irradiated in a closed water-circulating system for 5 h. The amount of evolved H_2_ was detected with an online TCD gas chromatograph (GC-7900, Techcomp, Shanghai, China), using nitrogen as the carrier gas. The stability of the samples was measured for 20 h with a 5 h run cycle.

The apparent quantum yield (*AQY*) for the evolution of H_2_ was calculated as follows:

(1) $$\eta_{AQY}=\frac{2M\times N_{A}\times h\times c}{S\times P\times t\times \lambda }\times 100\%$$

where *M* is the amount of H_2_ produced in the reaction (mol), *N_A_* is Avogadro’s constant (6.02 × 10^23^/mol), *h* is the Planck constant (6.626 × 10^−34^ J S), *c* is the vacuum light velocity (3 × 10^8^ m/s), *S* is the irradiation area (cm^2^), *P* is the monochromatic light intensity (W/cm^2^), *t* is the photoreaction time (s) and *λ* is the wavelength of the monochromatic light (m).

A characterization of the materials, photoelectrochemical and electrochemical measurements, and an evaluation of the amount of •OH samples produced are described in the supporting information.

## 3. Results and Discussion

**Figure 1:**
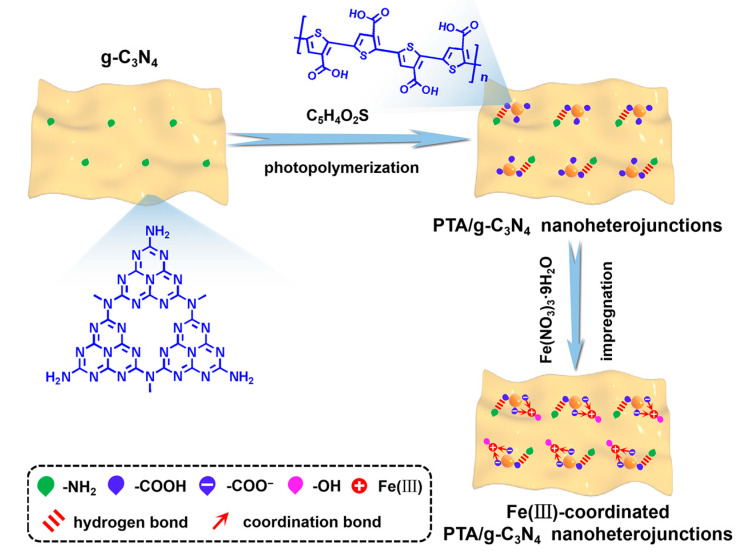
Schematic diagram of Fe(III)-coordinated PTA/g-C_3_N_4_ nanoheterojunctions.

The fabricating strategy and synthetic route for the Fe(III)-coordinated PTA/g-C_3_N_4_ nanoheterojunctions are shown in Figure 1 and Scheme S1. G-C_3_N_4_ nanosheets were first prepared by the thermal polymerization of urea. Subsequently, TA was linked to the g-C_3_N_4_ nanosheets by a hydrogen bond interaction between the -COOH of PTA and the -NH_2_ of g-C_3_N_4_. The tightly contacted PTA/g-C_3_N_4_ nanoheterojunctions were then constructed by the in situ photopolymerization of TA under irradiation with visible light. Finally, Fe(III) was introduced by coordinating with the -COOH of PTA to fabricate Fe(III)-coordinated PTA/g-C_3_N_4_ nanoheterojunctions, which are expected to be helpful for improving photogenerated charge separation and visible-light photocatalytic H_2_ evolution activities.

### 3.1. Structure Characterization

**Figure 2:**
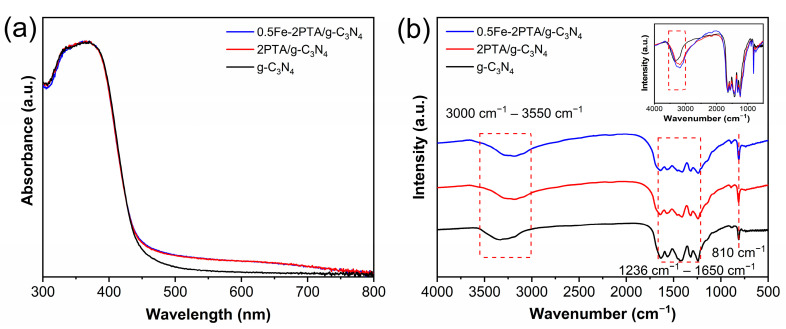
(**a**) UV–Vis diffuse reflection spectra and (**b**) FT-IR patterns of g-C_3_N_4_, 2PTA/g-C_3_N_4_ and 0.5Fe-2PTA/g-C_3_N_4_ (inset shows comparison of three FT-IR curves).

As presented in Figure S1a, it is clear that g-C_3_N_4_ displays two characteristic diffraction peaks at around 13.0° and 27.4°, corresponding to its (001) and (002) facets (JCPDS 87-1526) [38]. Additionally, PTA shows a wide peak at 24.9° (Figure S1b), which is attributed to the *π*-*π* stacking of the conjugate polymerization backbone [39]. By comparison, PTA/g-C_3_N_4_ and 0.5Fe-2PTA/g-C_3_N_4_ display similar diffraction peaks, indicating that the lattice structure of g-C_3_N_4_ does not change after coupling with PTA and coordinating with Fe(III) [21]. Notably, no apparent diffraction peaks of PTA are observed in any nanoheterojunctions, which can be ascribed to the low crystallinity and content of PTA [40]. The UV–Vis diffuse reflection (UV–Vis DRS, Shimadzu, Kyoto, Japan) spectra (Figure 2a) show that the light absorption range of 2PTA/g-C_3_N_4_ is expanded compared to g-C_3_N_4_, and there is no further change after coordinating with Fe(III), indicating that the introduction of Fe(III) does not affect the optical absorption. As shown in Figure S2, with the increase in the PTA content, the light absorption intensity of the xPTA/g-C_3_N_4_ in the visible light range clearly shows a gradual enhancement. It fully demonstrates that the visible-light response of g-C_3_N_4_ is significantly expanded due to the visible photosensitization effect of the introduced PTA.

To investigate the interface interactions between PTA and g-C_3_N_4_ in PTA/g-C_3_N_4_, Fourier-transform infrared (FTIR, Bruker, Karlsruhe, Germany) spectra of the samples were analyzed, as shown in Figure 2b and Figure S3. As shown in Figure S3a, PTA shows the carboxylic O-H and C=O stretching peaks at 3400 and 1610 cm^−1^, C-H stretching peaks at 3098, 2965 and 2915 cm^−1^, thiophene ring vibration peaks at 1533, 1437 and 1356 cm^−1^ and the C-S stretching vibration peak of the thiophene ring at 902 cm^−1^ [41,42,43,44]. It is worth noting that PTA presents a substantial absorption peak at 788 cm^−1^, corresponding to C*_β_*-H out-of-plane vibration, indicating that the *α*-*α* coupling is the dominant form of C-C bonding for the as-prepared PTA and there is almost no *α-β* coupling form [45]. As illustrated in Figure 2b, g-C_3_N_4_ shows a broad peak at around 3000–3550 cm^−1^, originating from the terminal -NH_2_. The peak at around 810 cm^−1^ is assigned to the hepazine ring bending vibration, and the set of peaks from 1236 cm^−1^ to 1650 cm^−1^ is ascribed to the C-N heterocycle stretching vibration [46,47]. By comparison, in xPTA/g-C_3_N_4_ (Figure 2b and Figure S3), the -NH_2_ absorption peak moves to a lower direction, indicating the interaction between the -COOH of PTA and the -NH_2_ of g-C_3_N_4_. Compared with 2PTA/g-C_3_N_4_, 0.5Fe-2PTA/g-C_3_N_4_ with coordinated Fe(III) presents an -NH_2_ absorption peak with no significant change in position, indicating that there may be no coordination bond interaction between the Fe(III) and -NH_2_ or that the interaction is negligible, which is consistent with another report [48]. Note that in Figure 2b, compared with that of g-C_3_N_4_, the peak intensity of 2PTA/g-C_3_N_4_ (at 3000–3550 cm^−1^) is strengthened due to the introduction of PTA with -COOH, and the peak intensity is further strengthened in 0.5Fe-2PTA/g-C_3_N_4_, attributed to the newly introduced Fe(III) with -OH [49].

**Figure 3:**
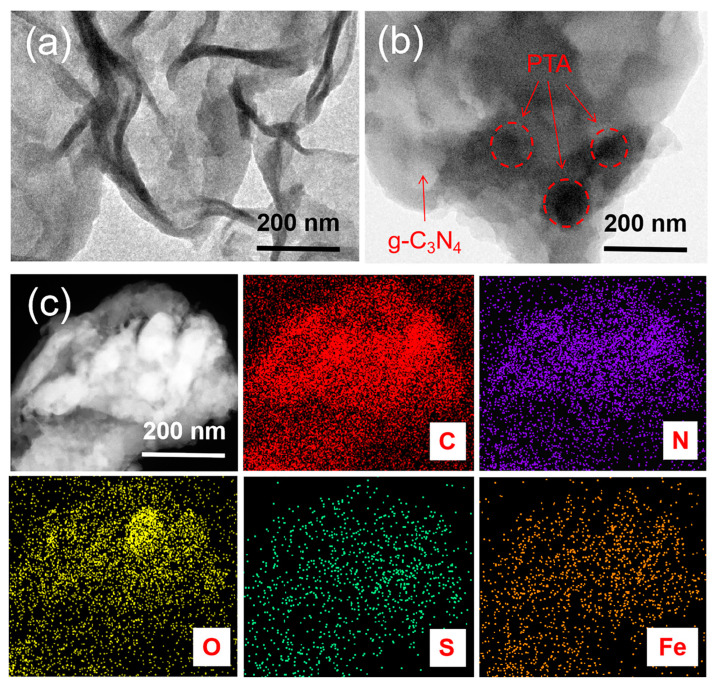
(**a**) TEM of g-C_3_N_4_. (**b**) TEM of 0.5Fe-2PTA/g-C_3_N_4_. (**c**) HAADF-STEM image and corresponding EDS mappings of 0.5Fe-2PTA/g-C_3_N_4_.

The morphology of the samples was studied using a scanning electron microscope (SEM, Hitachi, Tokyo, Japan). Figure S4a show that g-C_3_N_4_ displays the laminar structure. Figure S4b–e show that for xPTA/g-C_3_N_4_, PTA nanoparticles are dispersed on g-C_3_N_4_. It can be further observed from the transmission electron microscope (TEM) images that g-C_3_N_4_ (Figure 3a) displays 2D nanosheets and in 0.5Fe-2PTA/g-C_3_N_4_, nano-sized PTA particles with a diameter of 60~80 nm are evenly attached to the nanosheet surface of g-C_3_N_4_. Additionally, no Fe aggregates can be observed on the g-C_3_N_4_ surface, indicating that Fe does not exist as particles or clusters (Figure 3b). Furthermore, the high-angle annular dark-field scanning transmission electron microscopy (HAADF-STEM) image and the corresponding EDS mappings of 0.5Fe-2PTA/g-C_3_N_4_ (Figure 3c) clearly show the distribution of C, N, O, S and Fe elements, verifying the successful synthesis of 0.5Fe-2PTA/g-C_3_N_4_. It is also noted that the Fe and S elements exhibit a similar distribution, confirming the coordination of Fe(III) on PTA.

**Figure 4:**
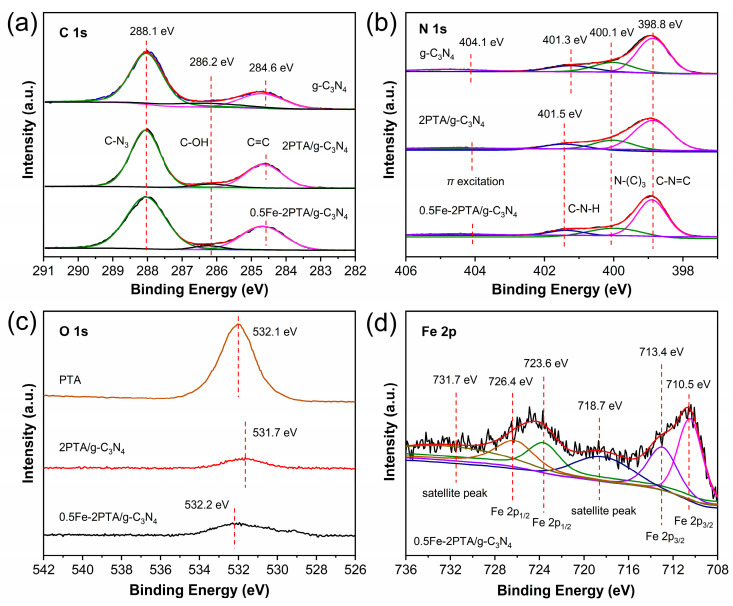
XPS spectra of samples: (**a**) C1s, (**b**) N1s, (**c**) O 1s and (**d**) Fe 2p.

In order to verify the interaction between the -COOH of PTA and the -NH_2_ of g-C_3_N_4_ or Fe(III), an X-ray photoelectron spectroscopy (XPS, Shimadzu, Kyoto, Japan) analysis was performed. As shown in Figure S5a, g-C_3_N_4_ is mainly composed of C and N, with a small amount of O coming from the adsorbed H_2_O, and PTA is mainly composed of C, N, O and S elements. Compared with 2PTA/g-C_3_N_4_, Fe was also detected in 0.5Fe-2PTA/g-C_3_N_4_ in addition to C, N, O and S, which is in good agreement with the result of the EDS mappings. The high-resolution XPS spectra were displayed to examine the chemical state of various elements. The C 1s spectra of g-C_3_N_4_, 2PTA/g-C_3_N_4_ and 0.5Fe-2PTA/g-C_3_N_4_ (Figure 4a) can be fitted into three peaks at 284.6 eV, 286.2 eV and 288.1 eV, attributed to C=C bonds in aromatic structures, the C-OH bond arising from some surface contamination and *sp*^2^ carbon atoms combined with N (C-N_3_) in the aromatic structure [50]. N 1s spectra of g-C_3_N_4_ (Figure 4b) can be fitted with four peaks at 398.8 eV, 400.1 eV, 401.3 eV and 404.1 eV, attributed to the *sp*^2^-hybridized aromatic N (C-N=C), the tertiary N bonded to C atoms in the form of N-(C)_3_, C-N-H side groups and *π* excitation, respectively [51,52]. It is noted that compared with that of g-C_3_N_4_, the C-N-H peaks of 2PTA/g-C_3_N_4_ and 0.5Fe-2PTA/g-C_3_N_4_ shift to the same higher binding energy (401.5 eV), indicating the decreased electron density around the N atoms of g-C_3_N_4_ in 2PTA/g-C_3_N_4_ and 0.5Fe-2PTA/g-C_3_N_4_ due to the hydrogen bond interaction between -NH_2_ of g-C_3_N_4_ and the -COOH of PTA and no obvious interaction between the N atom of g-C_3_N_4_ and the Fe(III) in 0.5Fe-2PTA/g-C_3_N_4_ [19,21].

As shown in Figure 4c, compared with that of PTA, the O 1s peak of 2PTA/g-C_3_N_4_ moves to a lower binding energy, indicating the increased electron density around the O atoms of PTA and confirming the hydrogen bond interaction between the -NH_2_ of g-C_3_N_4_ and the -COOH of PTA, combined with the above result of the C-N-H peak shift (Figure 4b). Interestingly, compared with that of the 2PTA/g-C_3_N_4_, the O 1s peak of 0.5Fe-2PTA/g-C_3_N_4_ exhibits a moderate shift to the higher binding energy direction, indicating the decreased electron density around the O atoms of PTA due to the coordination bond interaction between the introduced Fe(III) and the -COOH of PTA. The interaction between the Fe(III) and the -COOH of PTA was also verified by the O 1s peaks of the PTA and 0.5Fe-PTA in Figure S5b, which clearly shows the O 1s peak shift of the PTA from 532.1 eV to 532.4 eV after introducing the Fe(III) [53]. It is worth noting that in Figure 4c and Figure S5b, the O 1s peaks of the 0.5Fe-2PTA/g-C_3_N_4_ and 0.5Fe-PTA are higher than those of the 2PTA/g-C_3_N_4_ and PTA, respectively, which is due to the -OH of the introduced Fe(III). The Fe 2p spectra of 0.5Fe-2PTA/g-C_3_N_4_ (Figure 4d) can be fitted with Fe 2p_3/2_ peaks at 710.5 eV and 713.4 eV and the corresponding symmetric peaks of Fe 2p_1/2_ at 723.6 eV and 726.4 eV, respectively, while the peaks at 718.7 eV and 731.7 eV are ascribed to the Fe(III) satellite peak [35,48]. These are characteristic of Fe^3+^, further confirming the existence of Fe(III) in 0.5Fe-2PTA/g-C_3_N_4_. To further explore the change of the Fe 2p peaks caused by the interaction between Fe(III) and the -COOH of PTA, the Fe 2p spectra of 0.5Fe-2PTA, 0.5Fe-g-C_3_N_4_ and 0.5Fe-2PTA/g-C_3_N_4_ were also analyzed. As shown in Figure S5c, compared with those of 0.5Fe-g-C_3_N_4_, the Fe 2p peaks of 0.5Fe-PTA move to a smaller direction, demonstrating that the interaction of Fe(III) with the -COOH of PTA results in an increase in the electron cloud density around the PTA’s Fe atoms [54]. Compared with the 0.5Fe-PTA, slightly more obvious shifts to a smaller direction are observed in 0.5Fe-2PTA/g-C_3_N_4_, which may be attributed to the influence of the hydrogen bonds between the -COOH of PTA and the -NH_2_ of g-C_3_N_4_.

Based on the above analysis, it can be concluded that the tightly contacted Fe-2PTA/g-C_3_N_4_ nanoheterojunctions were successfully developed through the hydrogen bond interaction between the -COOH of PTA and the -NH_2_ of g-C_3_N_4_ as well as the coordination bond interaction between the Fe(III) and -COOH of PTA. The tight interface contact and the coordinated Fe(III) are expected to facilitate the transfer and separation of photogenerated charges in heterojunctions.

### 3.2. Photophysical and Photochemical Properties

**Figure 5:**
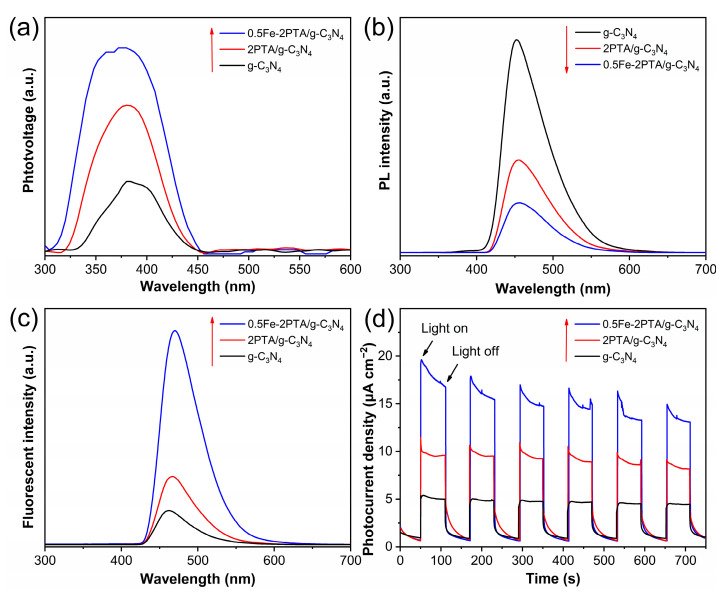
Photophysical and photochemical properties of samples. (**a**) SS-SPS responses in air. (**b**) PL spectra. (**c**) Fluorescence spectra related to the formed •OH amounts after irradiation for 1 h under irradiation with visible light. (**d**) Photocurrent response. (The arrows represent the changing tendency of the response signals.).

The photogenerated charge separation of the samples were first investigated by photophysical testing with steady-state surface photovoltage spectroscopy (SS-SPS, home-built, Harbin, Heilongjiang, China) and photoluminescence (PL, Shimadzu, Kyoto, Japan) measurements. In general, the higher the SS-SPS signal intensity, the more efficient the photogenerated charge separation. As shown in Figure 5a, a relatively low SS-SPS signal is observed on g-C_3_N_4_ due to the rapid photogenerated charge recombination, and 2PTA/g-C_3_N_4_ shows a more obvious SS-SPS signal than g-C_3_N_4_. In comparison, the SS-SPS signal of 0.5Fe-2PTA/g-C_3_N_4_ further increases, indicating its greatly improved photogenerated charge separation [55]. Moreover, as shown in Figure S6, SS-SPS signals first increase and then decrease with an increase in PTA content, and 2PTA/g-C_3_N_4_ shows the highest signal, meaning that the shortage of or excessive PTA content is not conducive to photogenerated charge separation. The PL spectra (Figure 5b) also show that 0.5Fe-2PTA/g-C_3_N_4_ demonstrates the lowest PL signal compared to 2PTA/g-C_3_N_4_ and g-C_3_N_4_, implying that the photogenerated charge recombination is greatly inhibited by constructing nanoheterojunctions with a tight interface contact and coordinating Fe(III) [26]. Furthermore, as shown in Figure S7, xPTA/g-C_3_N_4_ exhibits a lower PL signal than g-C_3_N_4_, and when the PTA content reaches 2 wt%, the highest photogenerated charge separation is obtained.

To further investigate the photogenerated charge separation in the photocatalytic reaction, the coumarin fluorescent measurement was carried out to detect the amount of formed •OH. A higher fluorescent signal corresponds to a higher amount of •OH, indicating a more efficient charge separation. As shown in Figure 5c, the dramatic fluorescence signal occurs on 2PTA/g-C_3_N_4_ and 0.5Fe-2PTA/g-C_3_N_4_ compared to g-C_3_N_4_, implying the improved photogenerated charge separation from the construction of nanoheterojunctions. Furthermore, the fluorescence signal of xPTA/g-C_3_N_4_ (Figure S8) is higher than that of g-C_3_N_4_, and when the PTA content reaches 2 wt%, the highest photogenerated charge separation is obtained [56]. The fluorescent intensity result is in agreement with the above SS-SPS and PL results. Figure 5d illustrates the periodic light on/off photocurrent response of the samples. It is obviously observed that the photocurrent intensity of 2PTA/g-C_3_N_4_ is higher than that of g-C_3_N_4_, and 0.5Fe-2PTA/g-C_3_N_4_ shows the highest photocurrent intensity, confirming the promoted photogenerated charge separation after constructing g-C_3_N_4_ nanoheterojunctions with PTA and further coordinating Fe(III). In addition, Nyquist plots of electrochemical impedance spectroscopy (EIS) (Figure S9) show that whether there is light or not, the arc radius of 0.5Fe-2PTA/g-C_3_N_4_ is the smallest compared to the arc radii of 2PTA/g-C_3_N_4_ and g-C_3_N_4_, suggesting an increased charge transfer, which helps with the photogenerated charge separation [57,58].

Based on the photophysical, photochemical and structural characterization results, it can be confirmed that constructing g-C_3_N_4_ nanoheterojunctions with an appropriate amount of PTA and further coordinating Fe(III) facilitate the photogenerated charge transfer and separation. Additionally, it is anticipated that 0.5Fe-2PTA/g-C_3_N_4_ will exhibit an enhanced photocatalytic performance.

### 3.3. Photocatalytic Activities for H_2_ Evolution

**Figure 6:**
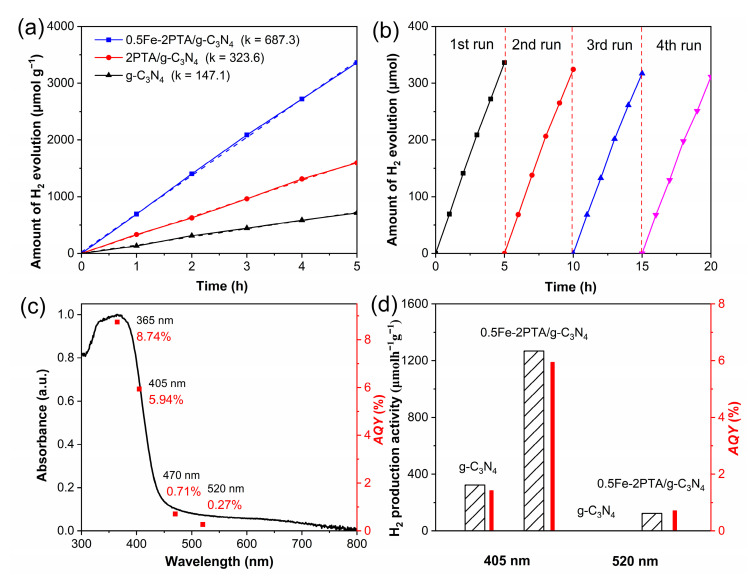
(**a**) Photocatalytic activities of water reduction for H_2_ evolution under irradiation with visible light. (**b**) Stability of H_2_ evolution amounts over 0.5Fe-2PTA/g-C_3_N_4_. (**c**) Wavelength-dependent *AQY* values and DRS spectrum of 0.5Fe-2PTA/g-C_3_N_4_. (**d**) Photocatalytic activities for H_2_ evolution at different excitation wavelengths (k is the rate constant of zero-order reaction for H_2_ production with the same meaning, unless stated elsewhere).

Photocatalytic activities were examined by detecting the amount of H_2_ evolution under illumination with visible light (*λ* > 420 nm), and the rate constant for H_2_ production was analyzed with the kinetic study. Figure 6a and Figure S10 manifest that the visible light photoactivity for H_2_ production follows zero-order kinetics. As shown in Figure 6a, g-C_3_N_4_ exhibits a meager photocatalytic H_2_ evolution activity of 147.1 µmol h^−1^ g^−1^, arising from the poor photogenerated charge separation. In comparison, 2PTA/g-C_3_N_4_ exhibits a noticeable increase in the photocatalytic H_2_ evolution activity, up to 323.6 µmol h^−1^ g^−1^ (~2.2 times that of g-C_3_N_4_). As expected, the photocatalytic H_2_ evolution activity of 0.5Fe-2PTA/g-C_3_N_4_ further increases, reaching up to 687.3 µmol h^−1^ g^−1^ (~4.6 times of g-C_3_N_4_) due to the significantly promoted charge separation. We also investigated the effect of PTA and Fe(III) content on the H_2_ evolution performance. As shown in Figure S10a,b, with the increase in the content of PTA or Fe(III), the H_2_ evolution activity first increases and then decreases. 2PTA/g-C_3_N_4_ and 0.5Fe-2PTA/g-C_3_N_4_ obtain the highest H_2_ evolution activity, respectively, meaning that excessive PTA or Fe(III) will hinder the improvement of the photogenerated charge separation and thus decrease the H_2_ evolution activities. The photocatalytic stability of 0.5Fe-2PTA/g-C_3_N_4_ was investigated for four cycles of 20 h irradiation. As shown in Figure 6b, the H_2_ evolution activity does not exhibit apparent deactivation around any cycle, confirming its considerable stability in the photocatalytic process. Figure 6c shows the wavelength-dependent apparent quantum yield (*AQY*) values and DRS spectrum of 0.5Fe-2PTA/g-C_3_N_4_. The *AQY* values of 8.74%, 5.94%, 0.71 % and 0.27% were obtained under the monochromatic irradiation of *λ* = 365, 405, 470 and 520 nm, respectively, which are similar to the changing tendency of its DRS curves. By comparison, the *AQY* values present superiority to some other reported g-C_3_N_4_ heterojunctions [24,29,59,60,61]. The photocatalytic H_2_ evolution activities at 405 nm and 520 nm excitation wavelengths were further investigated, as shown in Figure 6d. Compared to g-C_3_N_4_, 0.5Fe-2PTA/g-C_3_N_4_ shows a prominent improvement for photocatalytic H_2_ evolution activities and *AQY* values at excitation wavelengths of 405 nm and 520 nm. It can also be seen that the photocatalytic H_2_ evolution activity of 0.5Fe-2PTA/g-C_3_N_4_ at an excitation of 405 nm is better than that at excitation at 520 nm, indicating that the excitation of g-C_3_N_4_ is critical for the improved photocatalytic H_2_ evolution activities.

In order to further confirm the role of -COOH in PTA, H_2_ evolution measurements of 2PTh/g-C_3_N_4_ and 0.5Fe-2PTh/g-C_3_N_4_ were carried out for comparison in which there was no -COOH on the main chain of PTh; therefore, there was no hydrogen bond or coordination bond interaction between the PTh and g-C_3_N_4_ or Fe(III). It can be seen in Figure S10c that the photocatalytic H_2_ evolution activity of 2PTh/g-C_3_N_4_ only increases by 11% compared to that of g-C_3_N_4_, attributed to the poor interface interaction and thus low photogenerated charge transfer and separation between PTh and g-C_3_N_4_. Moreover, the photocatalytic H_2_ evolution activity of 0.5Fe-2PTh/g-C_3_N_4_ does not increase further, showing that the introduction of Fe(III) has no positive effect on 2PTh/g-C_3_N_4_. By comparison, 2PTA/g-C_3_N_4_ and 0.5Fe-2PTA/g-C_3_N_4_ exhibit obviously enhanced H_2_ evolution activities. This fully demonstrates that the -COOH of PTA interacts with the -NH_2_ of g-C_3_N_4_ by the hydrogen bond interaction and coordinates with Fe(III), which greatly improves the photocatalytic H_2_ evolution activities.

### 3.4. Discussion on Mechanism

**Figure 7:**
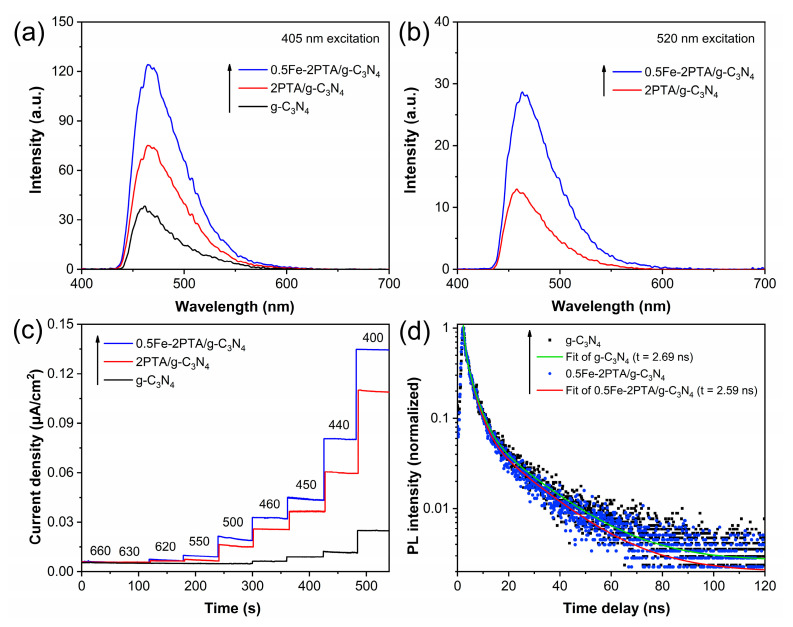
Fluorescence spectra related to the formed •OH amounts (**a**) at 405 nm excitation wavelength and (**b**) at 520 nm excitation wavelength. (**c**) Single-wavelength photocurrent action spectra under different excitation wavelengths. (**d**) TR-PL spectra excited under 369 nm.

To further verify the promoted photogenerated charge transfer and separation of the constructed nanoheterojunctions, the wavelength-dependent fluorescence spectra related to the formed •OH amounts of samples with monochromatic light excitation at 405 and 520 nm were performed, based on the results of Figure 6d. As shown in Figure 7a, under excitation at 405 nm, the strong signal of 0.5Fe-2PTA/g-C_3_N_4_ is observed, and its intensity is obviously higher than those of g-C_3_N_4_ and 2PTA/g-C_3_N_4_, indicating the significantly improved photogenerated charge separation [8]. And in Figure 7b, under excitation at 520 nm, no response signal is detected in g-C_3_N_4_, and 0.5Fe-2PTA/g-C_3_N_4_ presents higher signal intensity than 2PTA/g-C_3_N_4_ because in this situation, only PTA is excited and the photogenerated charge transfer occurs from PTA to g-C_3_N_4_. Noticeably, the signal intensities of 0.5Fe-2PTA/g-C_3_N_4_ and 2PTA/g-C_3_N_4_ under excitation at 520 nm are lower than those under excitation at 405 nm, illustrating that their photocatalytic H_2_ evolution activities, and *AQY* values under excitation at 520 nm are lower than those under excitation at 405 nm in Figure 6d. The single-wavelength photocurrent tests were also employed to investigate the photogenerated charge transfer and separation. Table S1 shows the highest occupied molecular orbital (HOMO) and LUMO position of g-C_3_N_4_ and PTA, which were determined from the UV–Vis DRS spectra (Figure S11) and cyclic voltammetry (CV) curves (Figure S12). The resulting optical energy gaps (*Eg*) of g-C_3_N_4_ and PTA are 2.70 eV and 1.98 eV, respectively (Figure S11). A detailed calculation method is shown as Method 1 in the Supporting Information. The optical absorption thresholds of g-C_3_N_4_ and PTA are determined to be about 460 and 626 nm, respectively, according to the equation *λ* = 1240/*Eg*. As depicted in Figure 7c, with the decrease of the excitation wavelength from 460 to 400 nm, the photocurrent density of g-C_3_N_4_ increases gradually. By comparison, 2PTA/g-C_3_N_4_ and 0.5Fe-2PTA/g-C_3_N_4_ exhibit enhanced photocurrent density from 620 nm to 460 nm, which is due to the visible photosensitization of PTA since in this situation, only PTA is excited [26]. It can be seen that under the irradiation wavelength of 440 nm, the photocurrent density of 2PTA/g-C_3_N_4_ increases sharply. According to the HOMO level position of g-C_3_N_4_ and the LUMO level position of PTA in Table S1, it can be calculated that 440 nm is the threshold wavelength for the excited high-energy electrons of g-C_3_N_4_ to the LUMO of the PTA. Therefore, it can be concluded that the sharp increase in the photocurrent density under 440 nm of irradiation is mainly attributed to the excited, high-level electron transfer from the LUMO of g-C_3_N_4_ to the LUMO of PTA, effectively promoting the photogenerated charge separation, and in this process, the slightly higher LUMO of PTA than that of g-C_3_N_4_ provides a high-energy electronic platform for the transferred electrons from g-C_3_N_4_. Moreover, an obviously larger photocurrent response is observed in 0.5Fe-2PTA/g-C_3_N_4_ than in 2PTA/g-C_3_N_4_, confirming that the coordinated Fe(III) can further facilitate the charge transfer and separation [48,53]. Time-resolved photoluminescence (TR-PL) measurements were also employed to investigate the photogenerated carrier lifetime and dynamic processes. It is accepted that a shortened decay lifetime indicates a more efficient photogenerated electron transfer [62]. As illustrated in Figure 7d, the decay lifetime of g-C_3_N_4_ is 2.69 ns, and the decay lifetime is shortened to 2.59 ns in 0.5Fe-2PTA/g-C_3_N_4_, indicating the photogenerated electrons of PTA and g-C_3_N_4_ could be extracted in a timely manner by constructing tightly contacted nanoheterojunctions dependent on the hydrogen bond and coordination bond interactions in 0.5Fe-2PTA/g-C_3_N_4_.

In addition, in order to further reveal the role of -COOH in the photogenerated charge transfer and separation of 0.5Fe-2PTA/g-C_3_N_4_, density functional theory (DFT) calculations with a Gaussian B3LYP/6-31G level were conducted to investigate the frontier molecular orbitals of PTA (Figure S13). It can be observed that the O atoms of -COOH in PTA participate in the HOMO and LUMO of PTA, which is advantageous for the rapid transfer and separation of photogenerated carriers between PTA and g-C_3_N_4_ through hydrogen bonds between the -COOH of PTA and the -NH_2_ of g-C_3_N_4_ [57].

Since Pt, as the cocatalyst, plays a very important role in the photocatalytic H_2_ evolution process, we further investigated the dispersion of deposited Pt on 0.5Fe-2PTA/g-C_3_N_4_. As shown in Figure S14a, the TEM image of 0.5Fe-2PTA/g-C_3_N_4_ with 1 wt% Pt loaded shows that Pt nanoparticles a few nanometers in size are distributed on the surface. They were also identified in the HRTEM image (Figure S14b) with a lattice spacing of 0.226 nm, matching the (111) crystal plane of Pt (JCPDS 04-0802). Furthermore, the HAADF-STEM and corresponding EDS mappings (Figure S14c) clearly show the uniform distribution of Pt on the surface, in addition to C, N, O, S and Fe elements. This is ascribed to the -COOH of PTA and -OH of Fe(III), which are favorable for the evenly dispersed Pt nanoparticles and thus beneficial to generating more active sites during the photocatalysis process and improving the H_2_ evolution activities.

**Figure 8:**
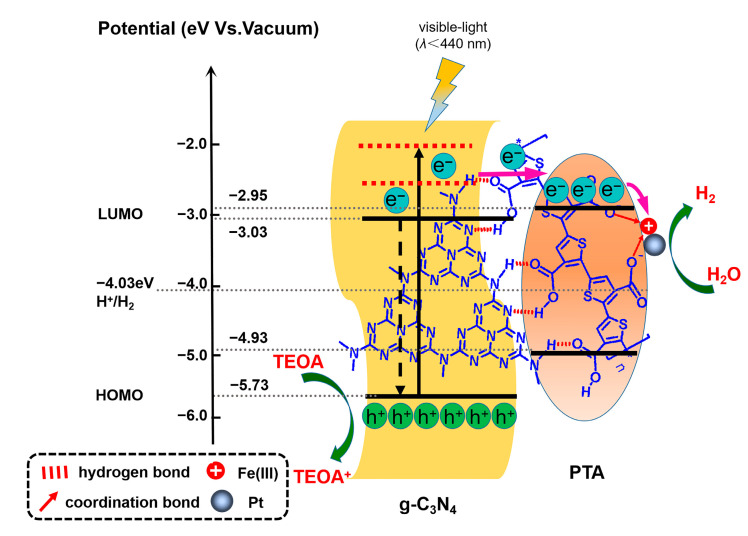
Mechanism of photogenerated charge separation and corresponding photochemical reactions in Fe(III)-coordinated PTA/g-C_3_N_4_ nanoheterojunctions.

Based on the above analysis, the possible mechanism of photogenerated charge transfer and separation was proposed in Figure 8 and Figure S14. It has been confirmed that 440 nm is the threshold wavelength for the high-level electron transfer (Figure 7c). As shown in Figure 8, under irradiation with visible light below 440 nm, although both the g-C_3_N_4_ and PTA in the nanoheterojunctions are excited simultaneously, in the nanoheterojunctions with g-C_3_N_4_ as the main part, the photogenerated charge separation enhancement mainly results from the high-energy electron transfer from the LUMO of g-C_3_N_4_ to the LUMO of PTA and the continuous transfer to the coordinated Fe(III) with -OH and a Pt cocatalyst. As shown in Figure S15, when the nanoheterojunctions are irradiated by visible light in a wavelength range from 460 nm to 620 nm, only PTA is excited: therefore, the photogenerated electrons of PTA transfer from the LUMO of PTA to the LUMO of g-C_3_N_4._ In this situation, PTA, as a photosensitizer, improves the utilization range of visible light.

## 4. Conclusions

In summary, using the in situ photopolymerization and impregnation methods, we have successfully constructed Fe(III)-coordinated poly(3-thiophenecarboxylic acid)/g-C_3_N_4_ nanoheterojunctions with tight interface contact for visible-light-driven H_2_ evolution. Compared to g-C_3_N_4_, the ratio-optimized 0.5Fe-2PTA/g-C_3_N_4_ exhibits a ~4.6-fold enhancement of visible-light photocatalytic H_2_ evolution activity, attributed to the significantly promoted photogenerated charge transfer and separation and the expanded visible-light response. It is proved that the tight interface contact, which is dependent on the hydrogen bond interaction between the -COOH of PTA and the -NH_2_ of g-C_3_N_4_ and the coordination bond interaction between Fe(III) and the -COOH of PTA, plays a key role in improving the photogenerated charge transfer and separation. It is also confirmed that the enhanced photoactivity is mainly ascribed to the high-energy electron transfer from the LUMO of g-C_3_N_4_ to the LUMO of PTA and the continuous transfer to the coordinated Fe(III) with -OH and a Pt cocatalyst in the nanoheterojunctions with g-C_3_N_4_ as the main part. This work not only provides a very feasible reference to design and synthesize efficient g-C_3_N_4_ heterojunction photocatalysts for visible-light-driven energy evolution but also helps us to understand the charge transfer and separation mechanisms in detail.

## Data Availability

The original contributions presented in the study are included in the article/Supplementary Material. Further inquiries can be directed to the corresponding author.

## Supplementary Materials

The following supporting information can be downloaded at: https://www.mdpi.com/article/10.3390/nano13081338/s1, Scheme S1: Synthetic route of Fe(III)-coordinated PTA/g-C_3_N_4_ nanoheterojunctions; Figure S1: (a) XRD patterns of g-C_3_N_4_, xPTA/g-C_3_N_4_ and 0.5Fe-2PTA/g-C_3_N_4_. (b) XRD pattern of PTA; Figure S2: UV–Vis diffuse reflection spectra of PTA, g-C_3_N_4_ and xPTA/g-C_3_N_4_; Figure S3: UV–Vis diffuse reflection spectra of PTA, g-C_3_N_4_ and xPTA/g-C_3_N_4_; Figure S4: SEM images of g-C_3_N_4_ (a) and xPTA/g-C_3_N_4_ (b–e). Figure S5: XPS spectra of samples: (a) survey and partially enlarged drawing, (b) O 1s and (c) Fe 2p; Figure S6: SS-SPS responses in air of g-C_3_N_4_ and xPTA/g-C_3_N_4_; Figure S7: PL spectra of g-C_3_N_4_ and xPTA/g-C_3_N_4_; Figure S8: Fluorescence spectra related to the formed •OH amounts of g-C_3_N_4_ and xPTA/g-C_3_N_4_ after irradiation for 1 h under visible-light irradiation (*λ* > 420 nm); Figure S9: Nyquist plots under visible light illumination (*λ* > 420 nm) of g-C_3_N_4_, 2PTA/g-C_3_N_4_ and 0.5Fe-2PTA/g-C_3_N_4_ (0.5 M Na_2_SO_4_ aqueous solution, pH = 7, SCE); Figure S10: Photocatalytic activities of water reduction for H_2_ evolution (1 wt% Pt-loaded, 10 vol% TEOA as sacrificial agent, *λ* > 420 nm) of samples: (a) g-C_3_N_4_ and xPTA/g-C_3_N_4_; (b) yFe-2PTA/g-C_3_N_4_; (c) comparison of g-C_3_N_4_, 2PTA/g-C_3_N_4_, 0.5Fe-2PTA/g-C_3_N_4_, 2PTh/g-C_3_N_4_ and 0.5Fe-2PTh/g-C_3_N_4_; Figure S11: UV–Vis spectra of g-C_3_N_4_ and PTA. Inset shows the Tauc plots of g-C_3_N_4_ and PTA; Figure S12: Cyclic voltammetry measurements of (a) g-C_3_N_4_ and (b) PTA; Figure S13: B3LYP/6-31G wave functions of the frontier molecular orbital in PTA and g-C_3_N_4_ with a chain length of n = 1. The grey ball: carbon atom; blue ball: nitrogen atom; red ball: oxygen atom; yellow ball: sulfur atom; white ball: hydrogen atom; Figure S14: (a) TEM image of 0.5Fe-2PTA/g-C_3_N_4_ (1 wt% Pt loaded). (b) HRTEM image of 0.5Fe-2PTA/g-C_3_N_4_ (1 wt% Pt loaded). (c) HAADF-STEM image and corresponding EDS mapping of 0.5Fe-2PTA/g-C_3_N_4_ (1 wt% Pt loaded); Figure S15: Mechanism of photogenerated charge separation and corresponding photochemical reactions in Fe(III)-coordinated PTA/g-C_3_N_4_ nanoheterojunctions; Table S1: HOMO and LUMO positions determined from CV curves, UV–Vis DRS spectra and DFT calculation methods. References [56,63,64] are cited in the Supplementary Materials.
