# Supplementary material for: Improved Visible-Light Photocatalytic H2 Evolution of G-C3N4 Nanosheets by Constructing Heterojunctions with Nano-Sized Poly(3-Thiophenecarboxylic Acid) and Coordinating Fe(III)
Source: Nanomaterials (Basel). 2023 Apr 12;13(8):1338. doi: 10.3390/nano13081338 (PMC10144103; doi:10.3390/nano13081338)
Supplement: Supplementary file 1 [file nanomaterials-13-01338-s001.zip › nanomaterials-2293987-supplementary.pdf]

nanomaterials

Supplementary Materials

Improved Visible-Light Photocatalytic H_2 Evolution of G-C_3N_4 Nanosheets by Constructing Heterojunctions with Nano-Sized Poly(3-Thiophenecarboxylic Acid) and Coordinating Fe(III)

Yong Li [1,2], Bingmiao Zhang [1], Xulong Pang [1], Zhijun Li [1], Yi Zhang [1], Ming Hao [1], Yan Zhu [1], Chuanli Qin [1,*] and Liqiang Jing [1]

[1] Key Laboratory of Functional Inorganic Material Chemistry (Ministry of Education), School of Chemistry and Materials Science, Heilongjiang University, Harbin 150080, China
[2] Engineering Research Center for Hemp and Product in Cold Region of Ministry of Education, Qiqihar University, Qiqihar 161006, China
* Correspondence: qinchuanli@hlju.edu.cn

1. Materials characterization and photoactivity evaluation

1.1. Materials Characterization

The infrared absorption spectra were investigated using a Fourier-transform infrared (FTIR) spectrophotometer (Equiox 55, Bruker, Karlsruhe, Germany). The morphologies and crystal phases of samples were characterized by transmission electron microscopy (TEM, JEM-2010, JEOL, Tokyo, Japan) and X-ray diffraction patterns (XRD, D8 Advance, Bruker, Karlsruhe, Germany) with a Cu Kα radiation source. High-angle annular dark-field scanning transmission electron microscopy (HAADF-STEM, JEM-2010, JEOL, Tokyo, Japan) and energy-dispersive X-ray spectroscopy (EDS, JEM-2010, JEOL, Tokyo, Japan) tests were operated on this TEM equipment. X-ray photoelectron spectroscopy (XPS, Shimadzu, Kyoto, Japan) was employed on a Kratos-Axis Ultra DLD apparatus equipped with an Al (monochromatic) X-ray source to investigate the surface element composition and chemical states, and binding energies were calibrated with respect to the signal for adventitious carbon (binding energy = 284.6 eV). UV–Vis diffuse reflectance spectra (UV–Vis DRS) were performed using a spectrophotometer (UV-2750, Shimadzu, Kyoto, Japan, with $BaSO_4$ as a reference). The photoluminescence (PL) spectra of samples were recorded using a spectrophotometer (Perkin-Elmer LS 55, Waltham, Massachusetts, USA) with an excitation wavelength of 325 nm. The steady-state surface photovoltage spectroscopy (SS-SPS, home-built, Harbin, Heilongjiang, China) measurements of the samples were obtained with a home-built apparatus equipped with a lock-in amplifier (SR 830) and synchronized with a light chopper (SR 540) at different atmospheres. The sample was sandwiched between two indium–tin–oxide (ITO) glass electrodes. A monochromatic light was obtained from 500 W Xe lamp (CHF XQ 500 W, Global Xe lamp power) through a double prism monochromator (SBP 300, Zolix, Beijing, China). Time-resolved photoluminescence (TR-PL) measurements were carried out on a single photon-counting spectrometer (EdinburghFLS 1000, Ediburgh Instruments, Edinburgh, Scotland, UK) with a radiation pulse of 369 nm, using a 50 ps pulse lamp. The fluorescence was detected at a wavelength of 420 nm.

1.2. Photoelectrochemical and Electrochemical Measurements

Photoelectrochemical and electrochemical measurements were carried out using an IVIUM V13806 electrochemical workstation in a traditional three-electrode system (coun-

ter electrode: Pt; reference electrode: saturated calomel electrode (SCE); working electrode: as-prepared sample film) with a 500 W Xe lamp as the light source in a 0.5 M Na_2SO_4 solution (pH = 7). Before each experiment, the solution was bubbled with high-purity nitrogen gas (99.999 %) for 20 minutes. Photocurrent response curves (I-t curves) of six cycle tests were obtained within 800 s under a 0.5 V bias. The photocurrent density tests were performed at different illumination wavelengths where the monochromatic illuminant was from a 500 W Xe lamp and a monochromator (CM 110). Electrochemical impedance spectroscopy (EIS) measurements were carried out with a 0.5 M Na_2SO_4 solution as the electrolyte, using the same three-electrode configuration over a frequency range from 10^2 to 10^5 Hz, with amplitude of 10 mV (Root Mean Square) and a bias of 0.5 V.

The cyclic voltammetry (CV) measurements were conducted using an IVIUM V13806 electrochemical workstation, using another three-electrode system (counter electrode: Pt; reference electrode: Ag/AgCl; working electrode: as-prepared sample film). The electrolyte was a tetrabutylammonium perchlorate aqueous solution (0.1 M, pH = 6) with a scanning rate of 50 mV s^{-1}.

The working electrode was prepared by the following method. A 100 mg sample was dissolved in 1.8 ml ethanol and 0.2 ml Nafion solution (5 wt%) and stirred for 5 days. Then the mixture was coated on the conductive surface of ITO glass and dried in a vacuum under 60 °C.

1.3. Evaluation of Produced •OH Amount

A 100 mg sample was dispersed in 50 ml coumarin solution (1×10^{-3} M). Prior to irradiation, the solution was stirred in the dark for 30 min to achieve the adsorption–desorption equilibrium. The sample was then irradiated for 1 h under visible light ($\lambda > 420$ nm) by a Xe lamp (150 W). After irradiation, the sample was centrifuged, and then the supernatant was tested using a spectrophotometer (Perkin-Elmer LS 55) to detect the fluorescence of 7-hydroxycoumarin with an emission peak at ~460 nm.

2. Scheme, Figures, Table, and Method

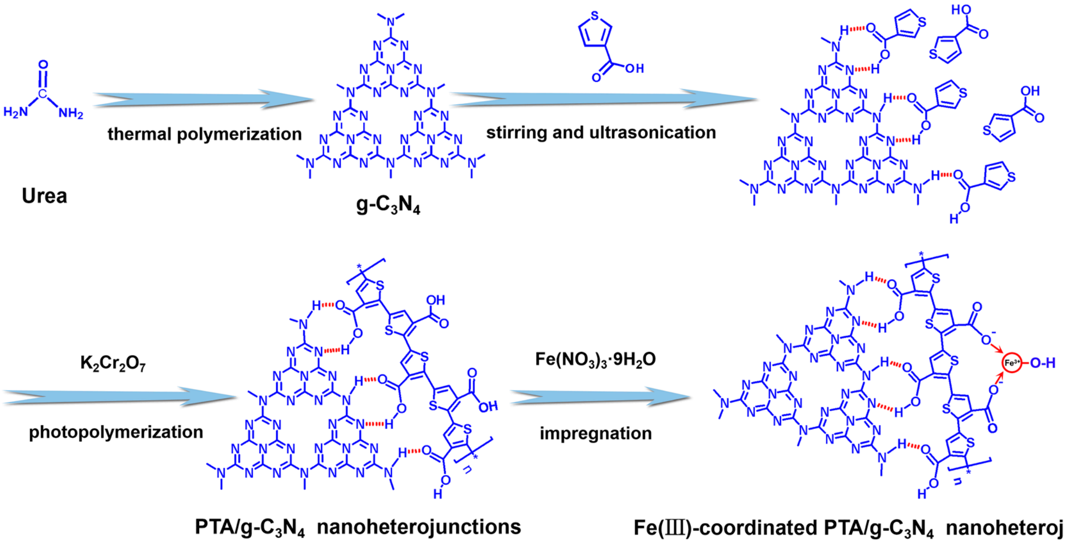

Scheme S1. Synthetic route of Fe(III)-coordinated PTA/g-C_3N_4 nanoheterojunctions.

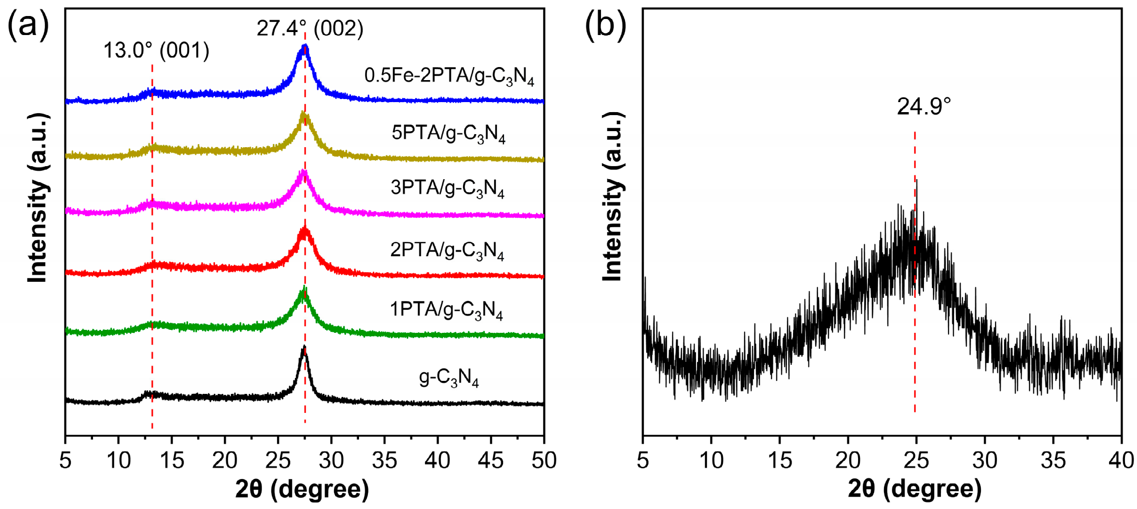

Figure S1. (**a**) XRD patterns of g-C_3N_4, xPTA/g-C_3N_4 and 0.5Fe-2PTA/g-C_3N_4. (**b**) XRD pattern of PTA.

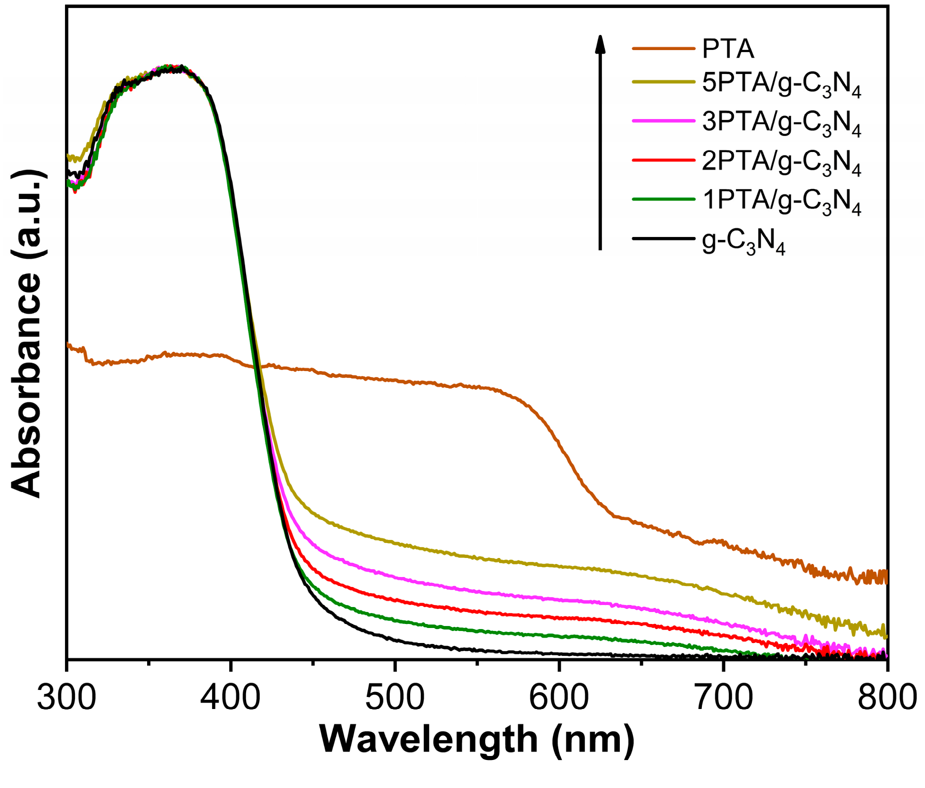

Figure S2. UV–Vis diffuse reflection spectra of PTA, g-C_3N_4 and xPTA/g-C_3N_4.

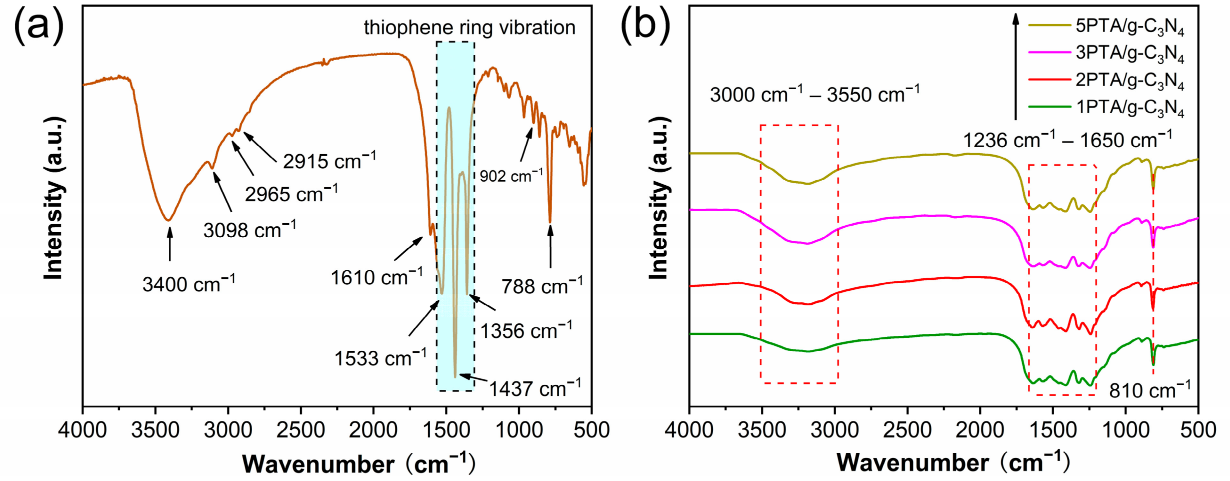

Figure S3. FT-IR spectra of PTA (**a**) and (**b**) xPTA/g-C_3N_4.

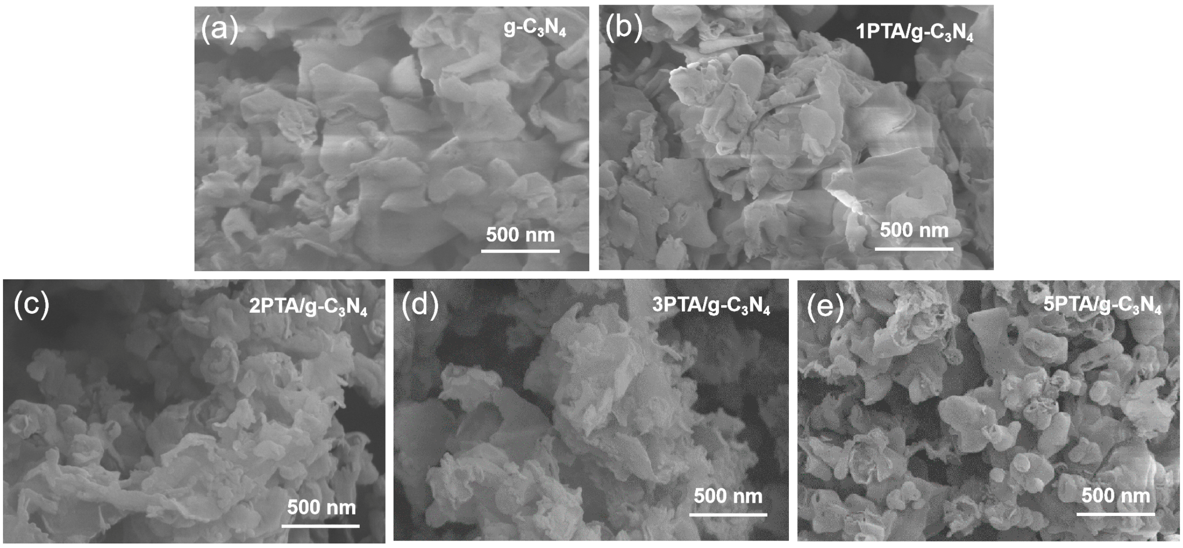

Figure S4. SEM images of g-C_3N_4 (**a**) and xPTA/g-C_3N_4 (**b**–**e**).

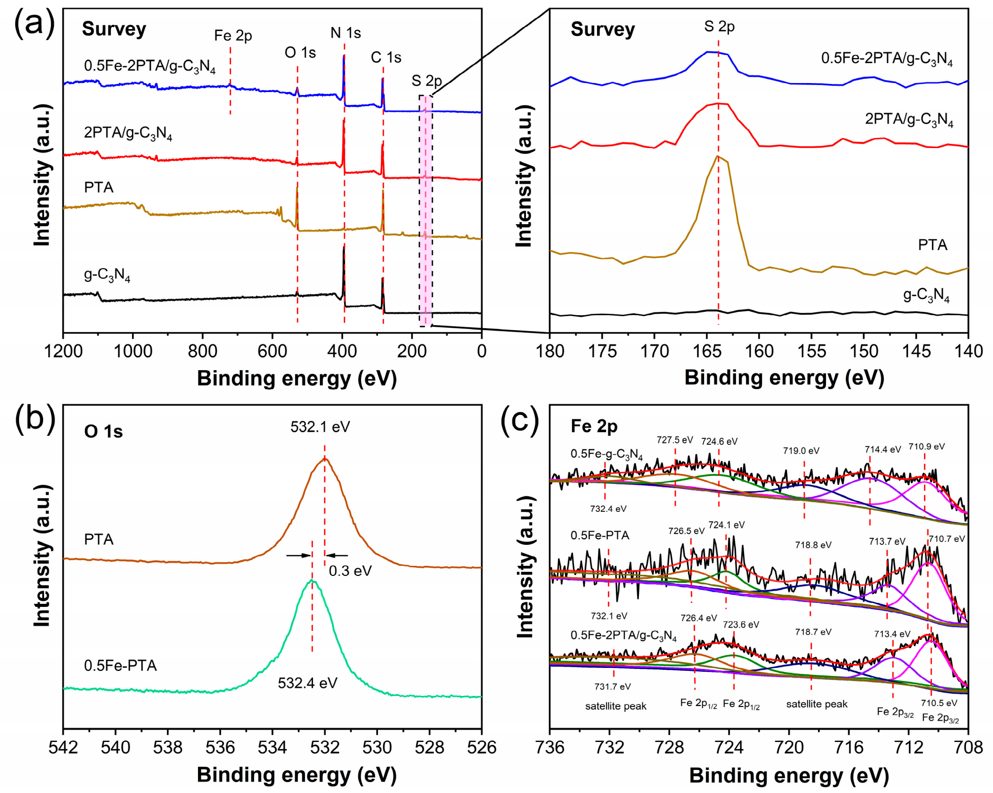

Figure S5. XPS spectra of samples: (**a**) survey and partially enlarged drawing, (**b**) O 1s and (c) Fe 2p.

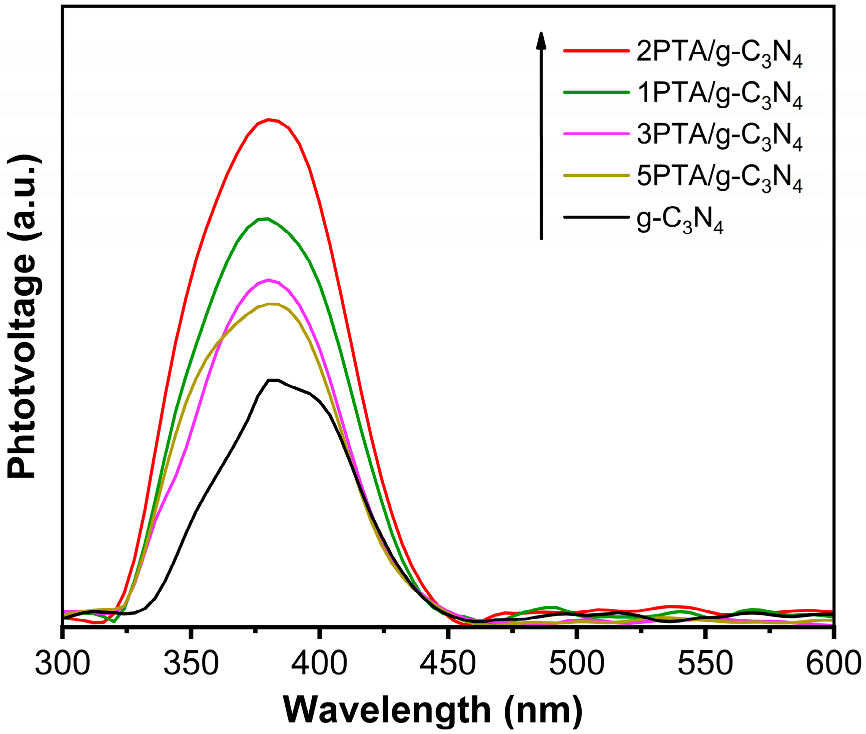

Figure S6. SS-SPS responses in air of g-C_3N_4 and xPTA/g-C_3N_4.

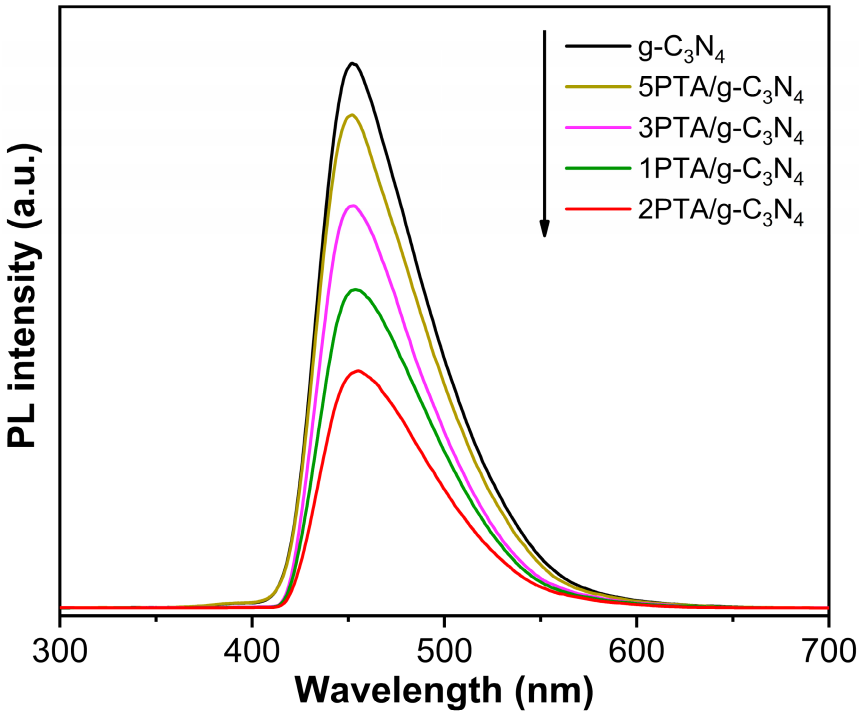

Figure S7. PL spectra of g-C_3N_4 and xPTA/g-C_3N_4.

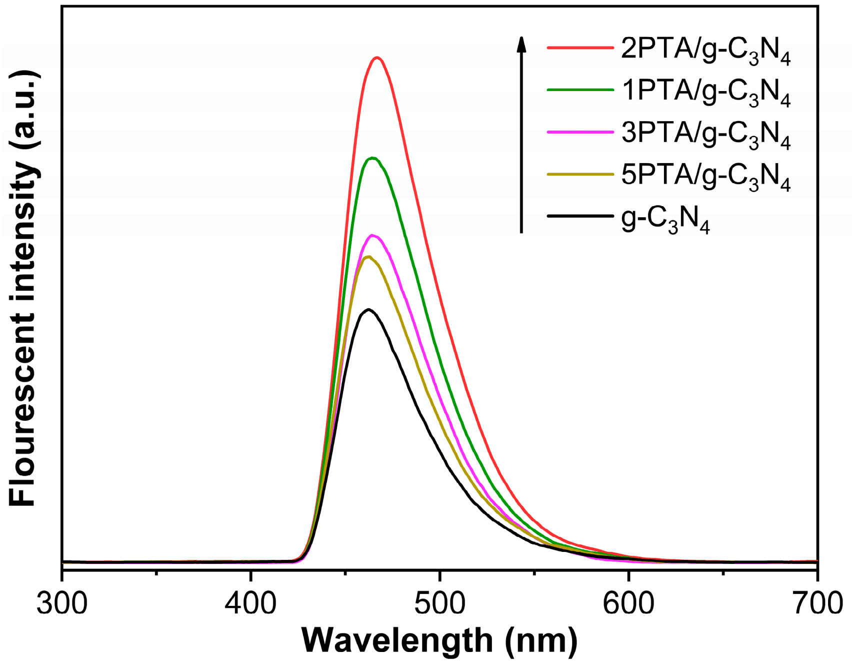

Figure S8. Fluorescence spectra related to the formed •OH amounts of g-C_3N_4 and xPTA/g-C_3N_4 after irradiation for 1 h under visible-light irradiation ($\lambda > 420$ nm).

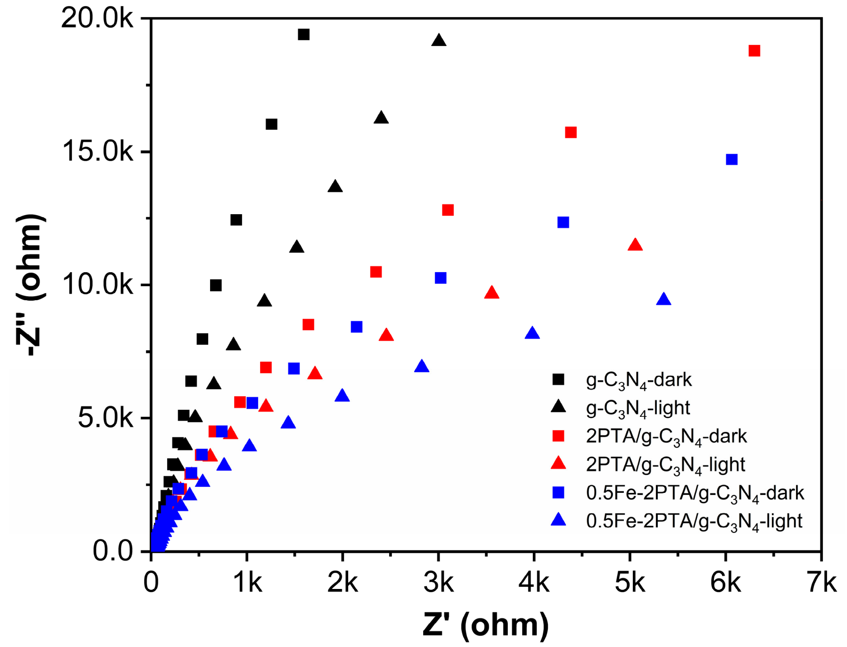

Figure S9. Nyquist plots under visible light illumination (λ > 420 nm) of g-C_3N_4, 2PTA/g-C_3N_4 and 0.5Fe-2PTA/g-C_3N_4 (0.5 M Na_2SO_4 aqueous solution, pH = 7, SCE).

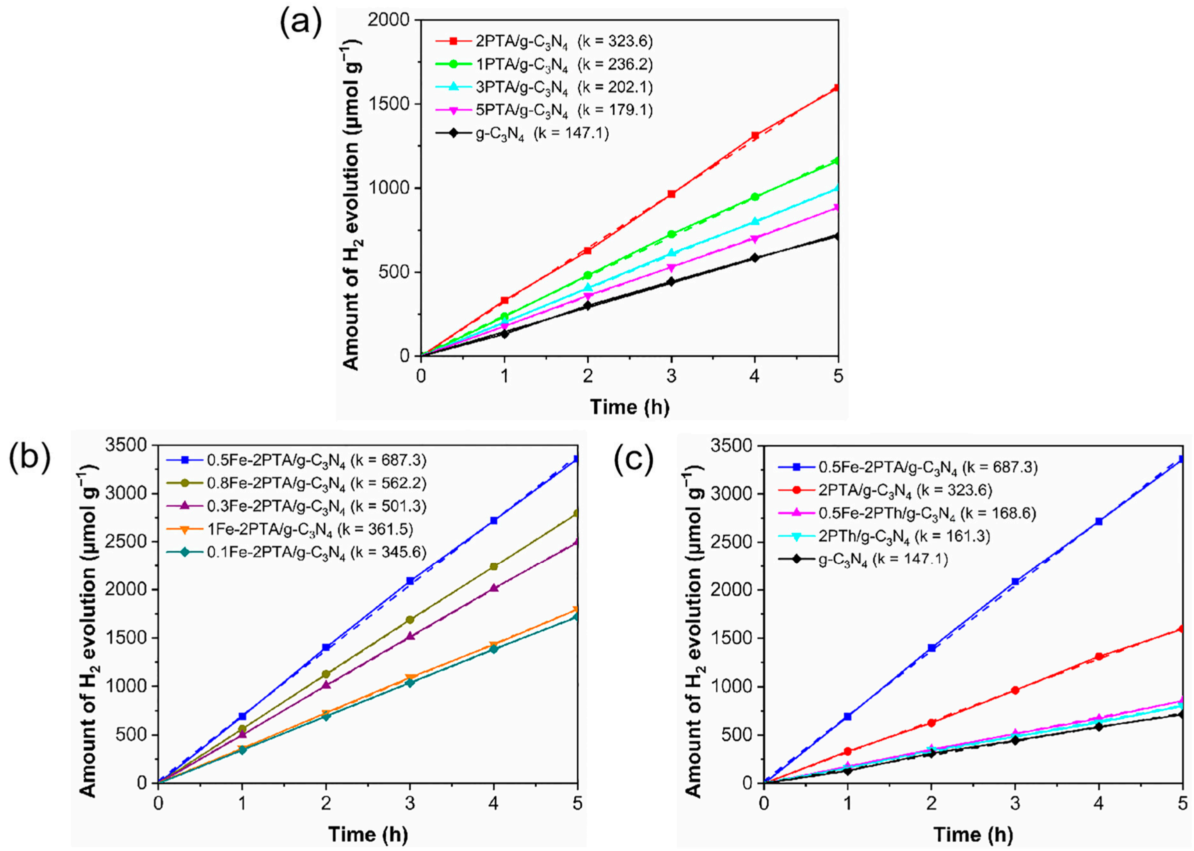

Figure S10. Photocatalytic activities of water reduction for H_2 evolution (1 wt% Pt-loaded, 10 vol% TEOA as sacrificial agent, λ > 420 nm) of samples: (**a**) g-C_3N_4 and xPTA/g-C_3N_4; (**b**) yFe-2PTA/g-C_3N_4; (**c**) comparison of g-C_3N_4, 2PTA/g-C_3N_4, 0.5Fe-2PTA/g-C_3N_4, 2PTh/g-C_3N_4 and 0.5Fe-2PTh/g-C_3N_4.

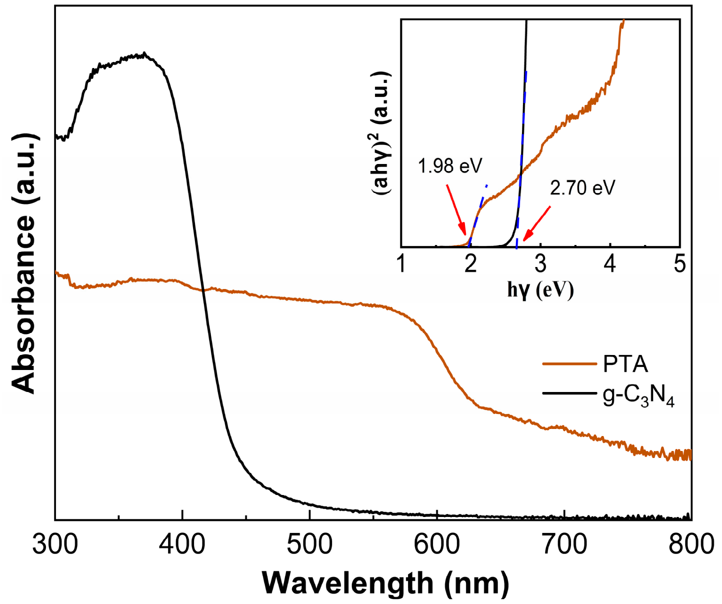

Figure S11. UV–Vis spectra of g-C_3N_4 and PTA. Inset shows the Tauc plots of g-C_3N_4 and PTA.

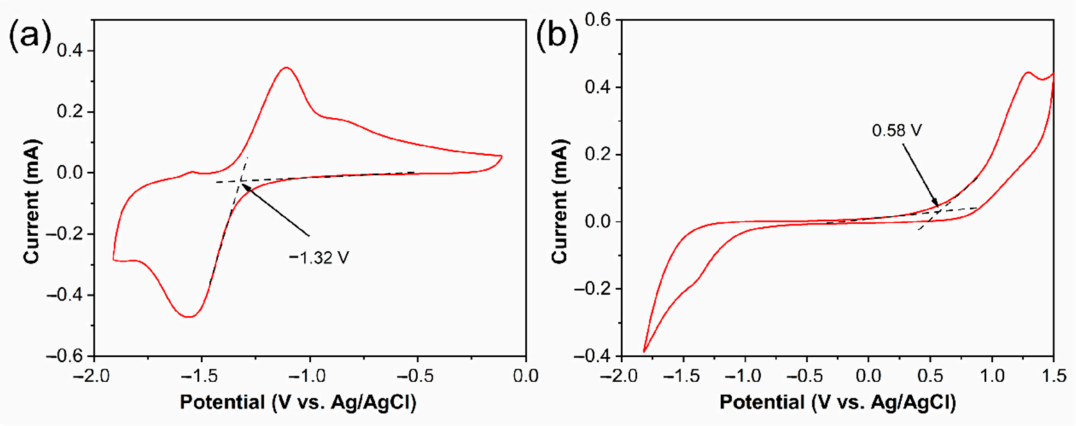

Figure S12. Cyclic voltammetry measurements of (**a**) g-C_3N_4 and (**b**) PTA.

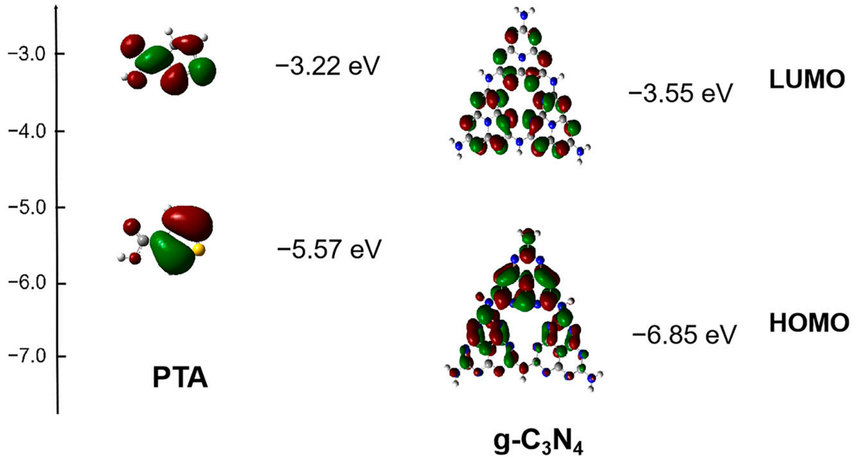

Figure S13. B3LYP/6-31G wave functions of the frontier molecular orbital in PTA and g-C_3N_4 with a chain length of n = 1. The grey ball: carbon atom; blue ball: nitrogen atom; red ball: oxygen atom; yellow ball: sulfur atom; white ball: hydrogen atom.

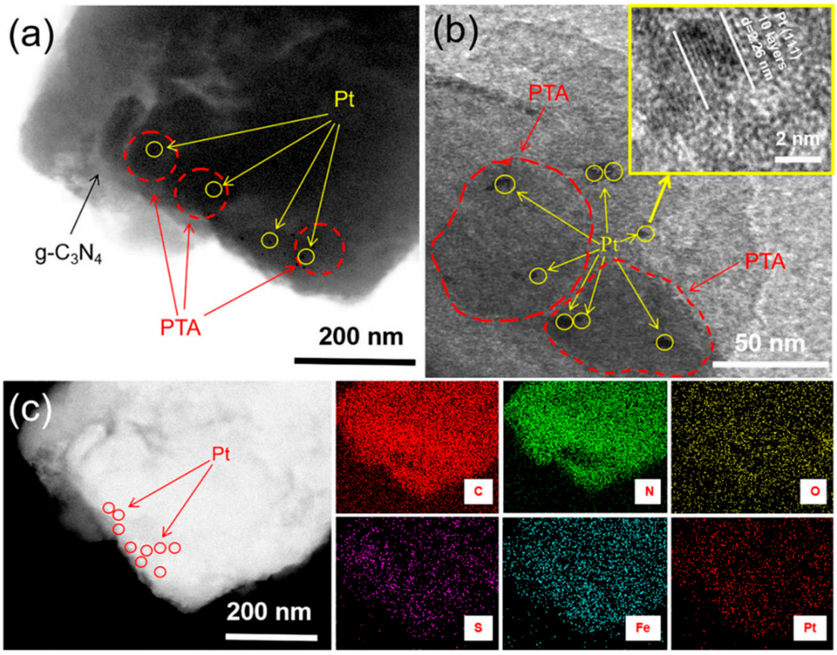

Figure S14. (**a**) TEM image of 0.5Fe-2PTA/g-C_3N_4 (1 wt% Pt loaded). (**b**) HRTEM image of 0.5Fe-2PTA/g-C_3N_4 (1 wt% Pt loaded). (**c**) HAADF-STEM image and corresponding EDS mapping of 0.5Fe-2PTA/g-C_3N_4 (1 wt% Pt loaded).

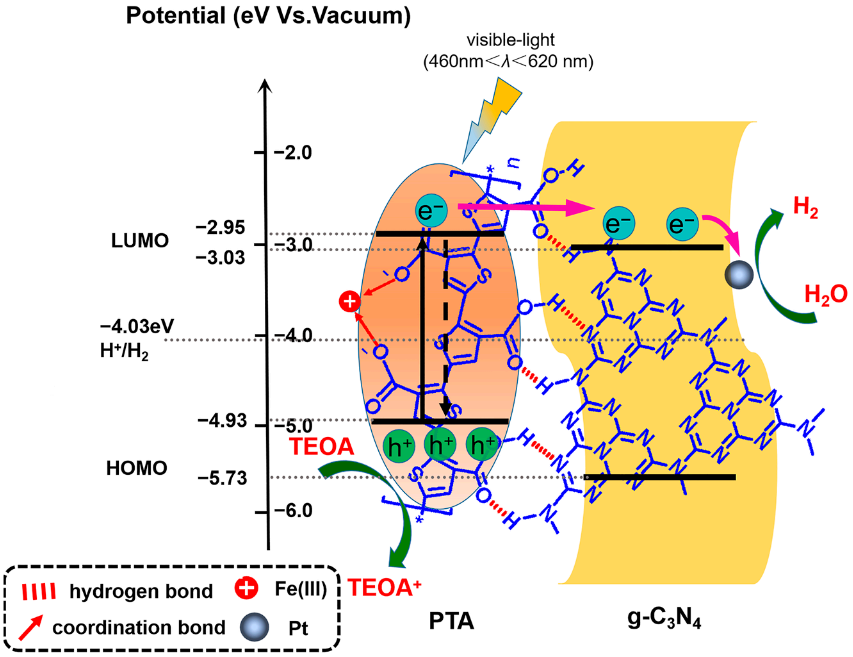

Figure S15. Mechanism of photogenerated charge separation and corresponding photochemical reactions in Fe(III)-coordinated PTA/g-C_3N_4 nanoheterojunctions.

Method 1:

The HOMO and LUMO were acquired from the UV–Vis DRS spectra (Figure S11) and cyclic voltammetry curves (Figure S12), according to the following method [1,2]:

Ferrocene was used as an external reference for calibration ($E_{1/2,\ ferrocene}$ = 0.45 V vs. Ag/AgCl). $E_{1/2,\ ferrocene}$ represents the average potential of the redox couple peaks of ferrocene. The HOMO of PTA and the LUMO of g-C_3N_4 are determined as follows:

$$E_{HOMO}\ (\mathrm{eV}) = -\ (E_{ox\_onset}\ \mathrm{vs.\ Ag/AgCl} + 4.80 - E_{1/2,\ ferrocene}) \quad (1)$$

$$E_{LUMO}\ (\mathrm{eV}) = -\ (E_{re\_onset}\ \mathrm{vs.\ Ag/AgCl} + 4.80 - E_{1/2,\ ferrocene}) \quad (2)$$

E_{ox_onset} and E_{re_onset} represent the onset oxidation potential of PTA and the onset reduction potential of g-C_3N_4 in the CV curves. The E_{ox_onset} of PTA and E_{re_onset} of g-C_3N_4 are marked with black arrows in Figure S12.

The optical absorption band edge (E_g) of PTA and g-C_3N_4 can be acquired according to the UV–Vis DRS spectra, as shown in the inset of Table S1 [3].

Table S1. HOMO and LUMO positions determined from CV curves, UV–Vis DRS spectra and DFT calculation methods.

Sample	CV				DFT		E_g (eV)
	E_{ox_onset} (V vs. Ag/AgCl)	E_{re_onset} (V vs. Ag/AgCl)	E_{HOMO} (eV vs. vacuum)	E_{LUMO} (eV vs. vacuum)	E_{HOMO} (eV vs. vacuum)	E_{LUMO} (eV vs. vacuum)	
g-C_3N_4	1.38	−1.32	−5.73	−3.03	−6.85	−3.55	2.70
PTA	0.58	−1.40	−4.93	−2.95	−5.57	−3.22	1.98

References

1. Zhou, W.; Jia, T.; Shi, H.; Yu, D.; Hong, W.; Chen, X. Conjugated polymer dots/graphitic carbon nitride nanosheet heterojunctions for metal-free hydrogen evolution photocatalysis. *J. Mater. Chem. A* **2019**, *7*, 303–311. https://doi.org/10.1039/C8TA09735F.
2. Jiang,L.; Yuan, X.; Zeng, G.; Wu, Z.; Liang, J.; Chen, X.; Leng, L.; Wang, H.; Wang, H. Metal-free efficient photocatalyst for stable visible-light photocatalytic degradation of refractory pollutant. *Appl. Catal. B: Environ.* **2018**, *221*, 715–725. https://doi.org/10.1016/j.apcatb.2017.09.059.
3. Li, Y.; Pang, X.; Zhao, Q.; Zhang, B.; Guo, X.; Zhang, Y.; Xie, Y.; Qin, C.; Jing, L. Controlled synthesis of nitro-terminated oligothiophene/crystallinity-improved g-C_3N_4 heterojunctions for enhanced visible-light catalytic H_2 production. *ACS Appl. Mater. Interfaces* **2023**, *15*, 5365–5377. https://doi.org/10.1021/acsami.2c21849.
